# Glycosyltransferase B4GALNT2 as a Predictor of Good Prognosis in Colon Cancer: Lessons from Databases

**DOI:** 10.3390/ijms22094331

**Published:** 2021-04-21

**Authors:** Michela Pucci, Nadia Malagolini, Fabio Dall’Olio

**Affiliations:** Department of Experimental, Diagnostic and Specialty Medicine (DIMES), General Pathology Building, University of Bologna, Via San Giacomo 14, 40126 Bologna, Italy; michela.pucci3@unibo.it (M.P.); nadia.malagolini@unibo.it (N.M.)

**Keywords:** glycosylation, colon cancer, Sd^a^ antigen, glycosyltransferases, epigenetic regulation

## Abstract

Background: glycosyltransferase B4GALNT2 and its cognate carbohydrate antigen Sd^a^ are highly expressed in normal colon but strongly downregulated in colorectal carcinoma (CRC). We previously showed that CRC patients expressing higher *B4GALNT2* mRNA levels displayed longer survival. Forced *B4GALNT2* expression reduced the malignancy and stemness of colon cancer cells. Methods: Kaplan–Meier survival curves were determined in “The Cancer Genome Atlas” (TCGA) COAD cohort for several glycosyltransferases, oncogenes, and tumor suppressor genes. Whole expression data of coding genes as well as miRNA and methylation data for *B4GALNT2* were downloaded from TCGA. Results: the prognostic potential of *B4GALNT2* was the best among the glycosyltransferases tested and better than that of many oncogenes and tumor suppressor genes; high *B4GALNT2* expression was associated with a lower malignancy gene expression profile; differential methylation of an intronic *B4GALNT2* gene position and miR-204-5p expression play major roles in *B4GALNT2* regulation. Conclusions: high *B4GALNT2* expression is a strong predictor of good prognosis in CRC as a part of a wider molecular signature that includes *ZG16*, *ITLN1*, *BEST2*, and *GUCA2B*. Differential DNA methylation and miRNA expression contribute to regulating *B4GALNT2* expression during colorectal carcinogenesis.

## 1. Introduction

**Figure 1:**
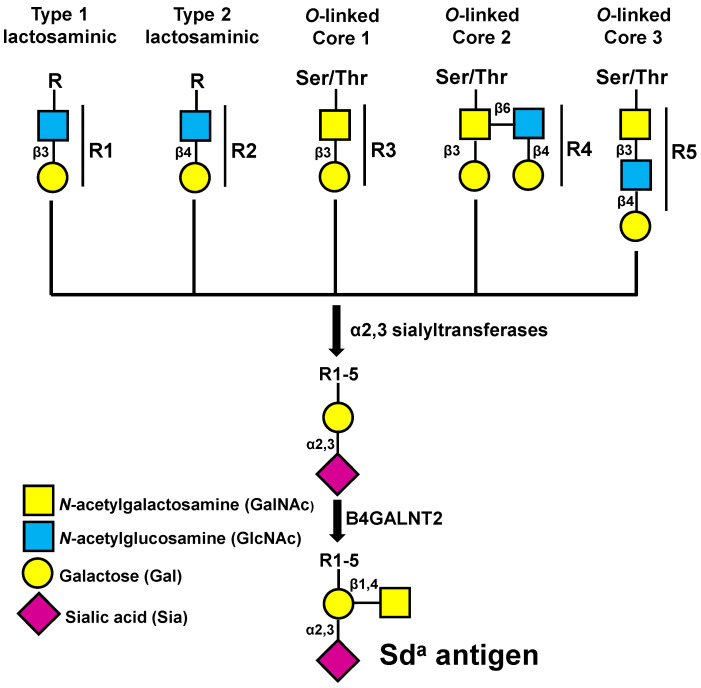
Schematic representation of the structure and biosynthesis of the Sd^a^ antigen. Several carbohydrate structures (R1–R5) terminating with galactose are α2,3 sialylated by different α2,3 sialyltransferases. The resulting sialylated structure is a substrate for B4GALNT2, which generates the Sd^a^ antigen.

Glycosylation plays a crucial role in a variety of biological processes, including intracellular transport, cell adhesion, cell growth, and apoptotic death [1]. In cancer, glycosylation changes consist of up- or downregulation of numerous carbohydrate structures affecting tumor invasion and progression [2,3,4]. The Sd^a^ antigen is a carbohydrate structure expressed on erythrocytes and a few other tissues in the vast majority of individuals. The minimal structure of the Sd^a^ antigen consists of a α2,3-sialylated galactose to which a GalNAc residue is β1,4-linked (Figure 1) [5]. This epitope can be carried out by various underlying sugar chains, which are indicated as R1–R5 in Figure 1. The enzyme responsible for the last step of Sd^a^ biosynthesis is β1,4*N*-acetylgalactosaminyltransferase 2 (B4GALNT2), first identified in our laboratory [6] and successively cloned in our and other labs [7,8,9]. The few Sd^a^-negative individuals display missense mutations in the C-terminal portion of the *B4GALNT2* gene [10]. The expression of *B4GALNT2* is very high in normal colonic mucosa but undergoes a dramatic downregulation in colorectal carcinoma (CRC) [11,12]. However, the level of expression in cancer samples is highly variable. In many specimens, it is undetectable, while it is relatively conserved in other cases, predicting a longer survival [13]. In mice, *B4galnt2* expression influences microbiota composition [14]. The *B4GALNT2* gene is comprised of 11 coding exons. There are two different alternative first exons, both containing a translational start codon. The polypeptide encoded by the transcript including Exon 1S (short form) contains a cytoplasmic tail of conventional length, whereas the one encoded by the transcript with Exon 1L (long form) possesses an exceptionally long cytoplasmic tail. The short form exhibits conventional Golgi localization, while the long form is localized in post-Golgi compartments as well as on the plasma membrane [15].

The molecular bases of *B4GALNT2* downregulation in colon cancer tissues and cell lines have not been fully elucidated, although methylation appears to plays a role. In fact, treatment of different colon cancer cell lines with the methylation inhibitor 5-aza-2′-deoxycytidine resulted in a partial activation of *B4GALNT2* expression [16,17]. The carbohydrate antigen sialyl Lewis^x^ (sLe^x^), a well-known ligand for the cell adhesion molecules of the selectin family, is ectopically expressed by many cancers and is associated with malignancy [2]. The sLe^x^ antigen is formed by a fucose residue α1,3-linked to the GlcNAc residue of a α2,3 sialylated type 2 chain, a structure on which B4GALNT2 acts to synthesize the Sd^a^ antigen [5]. In cell lines, the forced expression of *B4GALNT2* resulted in Sd^a^ expression and sLe^x^ inhibition, demonstrating that biosynthesis of the two antigens is mutually exclusive [12,18]. These *B4GALNT2*-expressing cell lines displayed reduced metastatic ability [18,19], which was attributed to sLe^x^ inhibition rather than to *de novo* Sd^a^ expression. However, our recent work [13,20] has shown that *B4GALNT2* expression decreases malignancy and stemness in different models of colon cancer cell lines, independently of sLe^x^ inhibition. The COADREAD (colon and rectal adenocarcinoma) cohort of “The Cancer Genome Atlas” (TCGA) contains data from 626 cases. Matched tumor–normal tissue samples have been molecularly characterized to identify alterations providing insights into the biology of CRC. Molecular data are derived from multiple types of analysis including gene expression, whole genome sequences, DNA methylation, and miRNA expression. In addition, numerous metadata, including clinical information about participants are also available. The purpose of the present study is to gain insights into the mechanisms of regulation of *B4GALNT2* expression and its association with malignancy through an in-depth analysis of data available in TCGA.

## 2. Results

### 2.1. The Level of Expression of Only a Few Oncogenes and Tumor Suppressor Genes Is Associated with Patient Survival

In a previous work, we reported that *B4GALNT2* expression is a significant predictor of long-term survival in a CRC TCGA cohort [13]. To put this observation into a more general context, we asked whether tumor suppressors and oncogenes known to play fundamental roles in cancer and, in particular, in CRC were better predictors of patient survival. In Figure S1, the Kaplan–Meier survival plots of CRC patients falling in the 15% upper or the 15% lower levels of several tumor suppressors and oncogenes are shown. The 15% threshold was chosen as a compromise between the need to focus on patients displaying extremely high or extremely low levels of gene expression and the need to include a sufficient number of patients to allow for statistical analysis. Survival curves have been ordered according to increasing *p*-value and boxed in red or blue according to the recognized tumor-promoting or tumor-suppressing activity of the genes, respectively. A statistically significant (*p* ≤ 0.05) association with survival was shown by genes *SMAD6*, *TERT*, *EGFR*, *CDKN2A*, *CTNNB1*, and *PIK3CA*, which encode the following molecules: *SMAD6*, an inhibitor of TGF-β signaling [21]; *TERT*, the reverse transcriptase subunit of telomerase; *EGFR*, the EGF receptor; *CDKN2A*, the cyclinD/CDK4,6 inhibitor p16^INK^ and the p53 activator p14^ARF^ through two partially overlapping open reading frames; *CTNNB1*, catenin-β1; and *PIK3CA*, the catalytic α subunit of phosphatidylinositol-4,5-bisphosphate 3-Kinase. For the tumor promoter genes *SMAD6*, *TERT*, *EGFR*, and *PIK3CA*, low levels of expression were associated with better survival, as expected. On the contrary, the longer survival associated with low expression of the *CDKN2A* locus was unexpected. On the other hand, low levels of catenin-β1 were associated with short-term poorer survival but with long-term survival. Genes for which the association with survival displayed *p*-values higher than 0.05 but lower than 0.1 included *CCNE1* (cyclin E1), *SMAD2*, *CDH1* (E-cadherin) *TP53*, and *BRAF* (B-Raf Proto-Oncogene, Serine/Threonine Kinase). Despite the established role of cyclin E1 in colon cancer promotion [22], long-term survival was associated with high *CCNE1* expression. On the other hand, the associations of *SMAD2*, *CDH1*, *TP53*, and *BRAF* with survival were consistent with their biological roles. These data reveal that the expression level of only a few oncogenes and tumor suppressor genes is associated with patients’ overall survival. A possible explanation for the lack of the expected relationships is provided by the fact that oncogene activation or tumor-suppressor inactivation can be due to mutations, altered phosphorylation, or mislocalization rather than increased or decreased mRNA expression.

### 2.2. Among Glycosyltransferases B4GALNT2 Has a Very Good Prognostic Value in CRC

**Figure 2:**
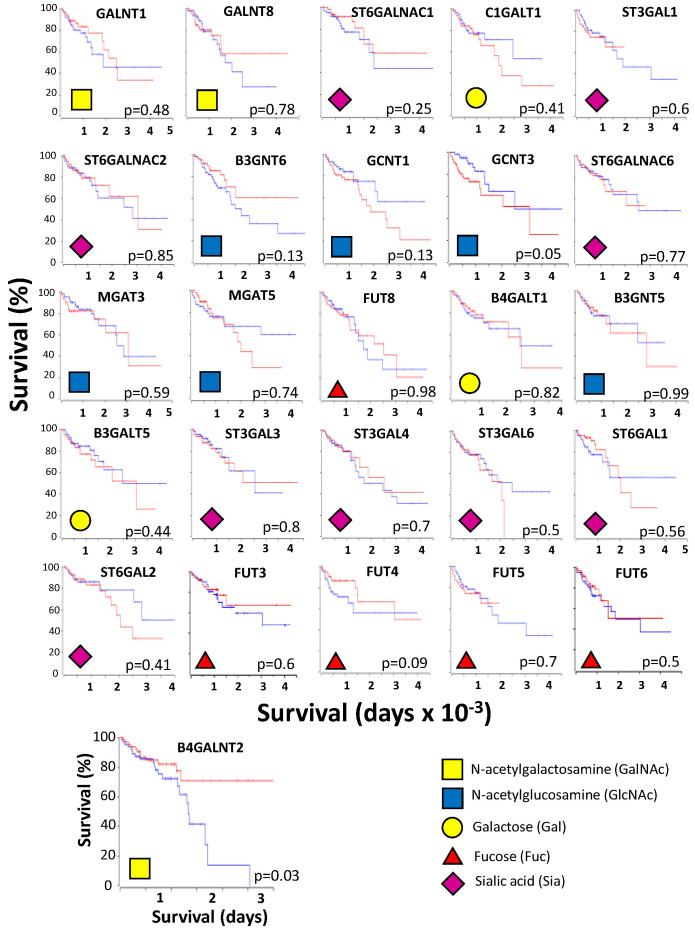
Kaplan–Meier survival plots of patients expressing high or low glycosyltransferase mRNA levels. Curves were generated by the OncoLnc website using the 15% higher (red lines) and 15% lower expressions (blue lines) of the indicated genes. The sugar transferred by each glycosyltransferase is indicated by the code in the insert.

Then, we investigated the relationship between patients’ survival and the expression of several glycosyltransferases relevant for the biosynthesis of cancer-associated carbohydrate structures. In Figure 2, the Kaplan–Meier survival curves of CRC patients falling in the 15 higher percentile and the 15 lower percentile of expression of the indicated glycosyltransferase genes are reported. Each insert contains the code for the sugar transferred by that glycosyltransferase. The enzymatic reactions in which the different glycosyltransferases are involved are reported in Figure S2. Among the 26 glycosyltransferases considered, *B4GALNT2* displayed the most significant association with prognosis (*p* = 0.03). In particular, long-term survivors belonged only to the high-expression group. *GCNT3*, the only other glycosyltransferase significantly associated with prognosis (*p* = 0.05), was previously identified as a member of a 15 glycosyltransferase prognostic signature in colon cancer [23]. However, none of the remaining 14 glycosyltransferases of that signature displayed a significant association with prognosis (data not shown). *GCNT1*, another GlcNAc transferase that shares with *GCNT3* the biosynthesis of *O*-linked Core 2 structures, displayed clearly, although not significantly, the same tendency for worse prognosis shown by *GCNT3*. This is consistent with the recognized association of Core 2 structures with the vessel invasion and depth of tumor invasion [24]. In contrast, *B3GNT6*, which synthesizes the Core 3 structure displayed a clear, although nonsignificant, tendency towards an opposite prognostic value, consistent with the recognized role of Core 3 structures in preventing colitis and colon cancer [25]. Surprisingly, enzymes responsible for the biosynthesis of well-known cancer-associated structures, such as β1,6 branching (*MGAT5*), sialyl-Tn (*ST6GALNAC1*) sLe^x^ (*FUT6*), Sia6LacNAc (*ST6GAL1*), and core-fucosylation (*FUT8*) lack any relationship with survival. Thus, among glycosyltransferases, *B4GALNT2* displays a very good prognostic value.

### 2.3. Comparison of High and Low B4GALNT2 Expressers in the COADRED Cohort

**Figure 3:**
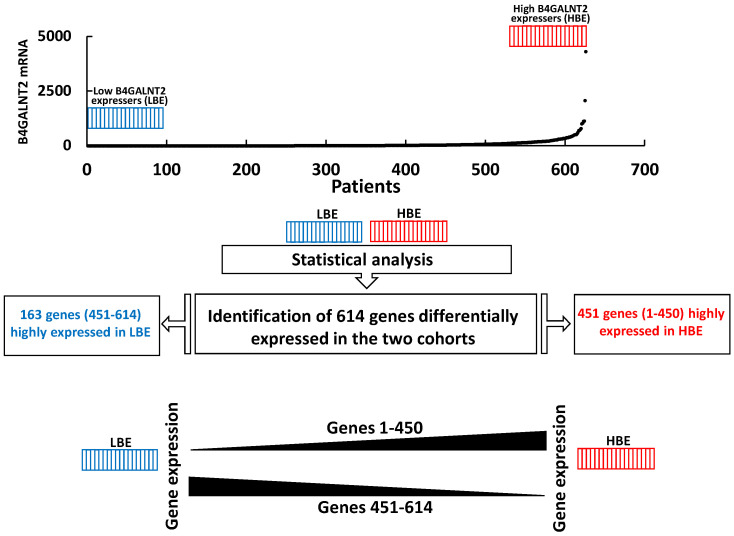
Schematic representation of the TCGA data analysis. The 626 COADREAD cases were ordered according to the level of B4GALNT2 expression, differentiating the 15% top expression (HBE, red) and the 15% lower expression (LBE, blue) cohorts of 94 cases. Statistical analysis (false discovery rate two-stage linear step-up procedure of Benjamini, Krieger, and Yekutieli) revealed that 614 genes were differentially expressed in the two cohorts. Of these, 451 genes were more expressed in HBE while 163 were more expressed in the LBE.

In the cohorts of the 15% higher B4GALNT2 expressers (HBE) and of the 15% lower B4GALNT2 expressers (LBE) the mean ± SD levels of *B4GALNT2* expression were 0 ± 0 and 367 ± 501, respectively. In total, 614 genes displayed a significantly different level of expression in the two cohorts (Figure 3 and Table S1). The vast majority (451) of these genes displayed higher expressions in HBE, although 163 genes displayed the opposite behavior. The gene expression ratio between high/low expressers ranged from 200 to −11. Genes showing the most remarkable changes, selected for a ratio higher than 10.0 or lower than −4.0, have been characterized by an extensive literature search, in particular for their role in cancer (Table 1). A color code was assigned to the putative tumor-promoting or tumor-restraining role in the change as follows: green for higher expression of tumor-restraining genes or lower expression of tumor-promoting genes in HBE and vice versa for red. Only genes with a recognizable role in cancer were reported. A high *B4GALNT2* expression was associated with 27 tumor restraining and 10 tumor promoting changes, suggesting its association with a low-malignancy molecular signature.

To establish the prognostic potential of genes modulated with respect to *B4GALNT2*, the survival curves of the top 15 highly expressed genes and the 10 least expressed genes in HBE were obtained (Figure S3). The predictive potential of the highly regulated genes was very good, while genes poorly expressed in HBE lacked any association with prognosis. In particular, the Kaplan–Meier curves of the 15 highly regulated genes (A) were relatively similar, with strong expresser patients displaying a more or less pronounced tendency towards better prognosis (the red curve is always above the blue curve). Four genes (*ZG16*, *ITLN1*, *BEST2*, and *GUCA2B*) displayed a statistically significant relationship. The significance of *ZG16*, a gene previously shown to be associated with a good prognosis in CRC [26], was particularly high. The *p*-value of these genes was always lower than 0.5, which could be indicative of a tendency, although not statistically significant. On the other hand, genes poorly expressed in HBE displayed *p*-values always above 0.5.

### 2.4. Several Glycogenes Are Differentially Modulated in HBE and LBE

In Table 2, genes encoding glycosyltransferases as well as heavily glycosylated glycoproteins, such as mucins, and sugar binding proteins, such as galectins, for which the expressions are significantly different in HBE and LBE are reported. The vast majority of the genes listed in Table 2 displayed higher expressions in HBE. In particular, we observed a 10-fold higher expression of *GALNT8*, mediating the first step of *O*-glycan biosynthesis (Figure S2), and a 14-fold higher expression of *B3GNT6*, the enzyme mediating the biosynthesis of Core 3 structures. Since the decrease in Core 3 structures is associated with colon cancer [35], the higher expression of *B3GNT6* is consistent with a colon cancer phenotype closer to normal. In addition, Core 3 structures are major Sd^a^ carriers in normal colon [36]. On the other hand, HBE displayed higher levels of expression of the sialyltransferases *ST6GALNAC1* and *ST6GALNAC2*, which are responsible for biosynthesis of the tumor-associated antigens sialyl-Tn and sialyl-6-T. Galactosyltransferase *B3GALT5* is a key enzyme in the biosynthesis of type 1 lactosaminic chains (Galβ1,3GlcNAc) highly expressed in normal colon but downregulated in CRC [37]. Its higher expression in HBE is consistent with an association with lower malignancy. α2,3-sialylation of type 2 chains, which is mediated (among other STs) by ST3GAL4, is a necessary step in Sd^a^ biosynthesis. Concomitantly high ST3GAL4 and B4GALNT2 expressions in the same tissue are expected to support a strong level of Sd^a^ biosynthesis. On the other hand, the 10-fold higher expression of *FUT5*, which mediates sLe^x^ biosynthesis and acts as a possible competitor of B4GALNT2, reduces Sd^a^ expression. *ST6GAL1* and *ST6GAL2*, which catalyze the α2,6-siaylation of glycoproteins [38] and oligosaccharides [39], are the only two glycogenes that display lower expressions in HBE. ST6GAL1 is overexpressed in CRC [40] and is associated with increased malignancy at the protein level [41] but not at the mRNA level [42].

### 2.5. The Role of Methylation in B4GALNT2 Expression

**Figure 4:**
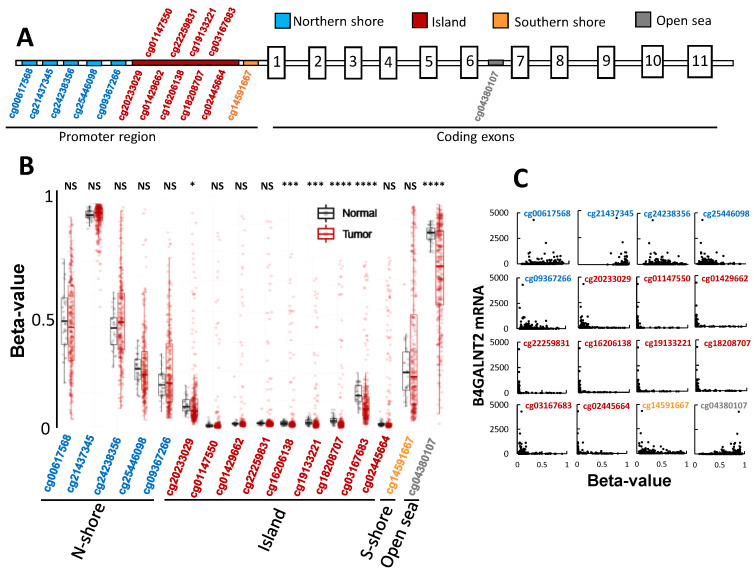
DNA methylation of the *B4GALNT2* gene. (**A**) Schematic representation, not drawn to scale, of the promoter region and of the coding exons of the *B4GALNT2* gene. The approximate position of the probes is indicated. (**B**) Methylation level of the different probes. * *p* < 0.05; *** *p* < 0.001; **** *p* < 0.0001. (**C**) Correlation between *B4GALNT2* expression level and methylation of specific positions in tumor tissues.

The presence of CpG islands in the promoter region of the *B4GALNT2* gene suggests that methylation can play a relevant role in *B4GALNT2* downregulation in colon carcinogenesis. The methylation probes used by the TCGA characterization covered the CpG island as well as a Northern shore (N-shore, upstream the island), a Southern shore (S-shore, downstream the island), and an intronic (open-sea) position between exons 6 and 7 (Figure 4A). At first sight, several differences were evident between normal and tumor methylation (Figure 4B). Which insights do these data provide into the role of methylation in the general downregulation of *B4GALNT2* mRNA observed in colon cancer? Methylation in both the N-shore and the S-shore was never statistically different between normal and tumor tissues, although in the latter the extent of methylation was more heterogeneous among patients (Figure 4B). Consequently, these changes cannot be responsible for the general *B4GALNT2* downregulation in cancer. Seven positions within the island (cg01147550-cg18208707 and cg02445664) displayed very low levels of methylation in both normal and the vast majority of tumor tissues, ruling out their major role in cancer-associated *B4GALNT2* downregulation. Positions cg20233029 and cg03167683 in the island displayed a small but significantly reduced methylation in tumor tissues. However, to explain the reduced *B4GALNT2* expression, methylation of these sites should be increased rather than decreased. Methylation of the “open sea” intronic site cg043380107 displayed a highly significant and very heterogeneous reduction in cancer. As shown in Figure 4C, which reports the correlation between *B4GALNT2* expression and methylation status of the 16 sites in tumor tissues, in some cases, methylation results in enhancement, rather than inhibition, of gene expression. This is particularly true for the intronic site cg043380107, in which the highest *B4GALNT2* expression levels are associated with high methylation. Except for the first two sites in the N-shore, in all of the remaining sites, low methylation is a prerequisite for high *B4GALNT2* expression, although many samples displaying very low methylation failed to express *B4GALNT2* (Figure 4C). Altogether, these data point to reduced methylation of the intronic site as a key factor in accounting for the general reduction of *B4GALNT2* expression in CRC and demonstrated that, among CRC samples, low methylation of the island and shore sites is a necessary but not sufficient condition for high *B4GALNT2* expression.

### 2.6. The Role of miRNAs in B4GALNT2 Expression

**Figure 5:**
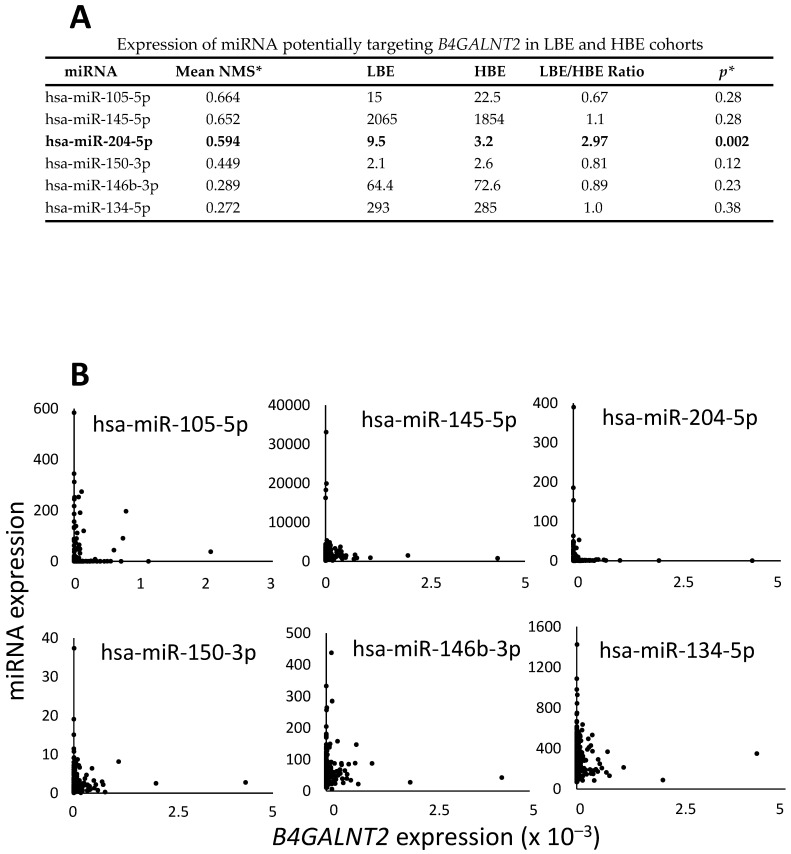
Correlation of *B4GALNT2* and miRNA expression. (**A**) miRNA potentially targeting *B4GALNT2* were obtained from the CSmiRTar database. Only miRNA supported by at least two of the four miRNA prediction target databases and with a “normalized miRNA score, NMR” >0.2 were considered. LBE and HBE represent the expression of the miRNA in the LBE (*B4GALNT2* expression = 0) or HBE (*B4GALNT2* expression closest to 367) cohorts. The only significantly modulated miRNA is indicated in bold. * Student’s t test for independent samples. (**B**) dot plot correlation analysis of the miRNA and *B4GALNT2* expression level.

The potential role of miRNAs in the regulation of *B4GALNT2* expression was investigated. Preliminarily, a list of miRNA potentially targeting *B4GALNT2* in colorectal cancer was obtained from the CSmiRTar database. Only miRNA supported by at least two of the four miRNA target prediction databases and with a “normalized miRNA score, NMR” >0.2 (Figure 5A) were considered. The mean level of miRNA expression was determined in the LBE and HBE groups. In consideration of the different number of TCGA patients for which miRNA data were available, we included in the LBE and HBE cohorts the top lower and top higher expressers displaying a mean level of *B4GALNT2* expression closest to that of the LBE and HBE cohorts shown in Figure 3 (0 and 367, respectively). Five miRNA displayed little and nonsignificant differences between LBE and HBE (Figure 5A). On the other hand, miR-204–5p was nearly 3-fold less expressed in HBE than in LBE (*p* = 0.002). A correlation dot plot (Figure 5B) revealed that miR-204–5p was never expressed in HBE patients, although several patients not expressing miR-204–5p failed to express *B4GALNT2*. These data strongly suggest that downregulation of miR-204–5p is a necessary but not sufficient condition for *B4GALNT2* expression in CRC tissues.

## 3. Discussion

In this study, we showed that *B4GALNT2* mRNA expression exhibits a prognostic predictive potential in CRC much better than that of all of the glycosyltransferases tested and even better that that of many oncogenes and tumor-suppressor genes. Among those tested, only *SMAD6* and *TERT* displayed better prognostic values than *B4GALNT2*, which equals that of *EGFR* (Figure S1). Patient stratification according to *B4GALNT2* expression revealed that HBE group displayed a concomitantly high level of other genes associated with positive prognosis, such as *ZG16*, *ITLN1*, *BEST2*, and *GUCA2B*. (Table 1). ZG16 is an animal homologue of a plant lectin [43] that inhibits the growth of colon cancer cell lines and CRC-derived organoids by binding with cell surface sugars [29]. In addition, it downregulates PD-L1 expression in CRC, promoting immune surveillance [27]. *ITLN1* is associated with good prognosis in CRC [30,32]. It encodes a lectin known as intelectin-1 or omentin-1, which acts as a tumor suppressor in CRC [31]. Apart from its association with good prognosis in CRC [33], no more information is available for *BEST2*. The product of the *GUCA2B* gene, in cooperation with those of *GUCA2A* and *GUCA2C*, regulates proliferation, metabolism, and barrier function in the intestine [44]. In long- and short duration ulcerative colitis, *GUCA2B* displays a differential expression in parallel with *B4GALNT2* [45]. Among the genes more dramatically downregulated in HBE, *TSIX* is of particular interest. It is a long noncoding RNA (lncRNA) known to be the anti-sense inhibitor of *XIST*, which is responsible for the epigenetic inactivation of one of the two X chromosomes in female cells. However, *TSIX* has also been shown to synergistically regulate, together with other lncRNAs, cancer genes and pathways across multiple tumor contexts [34]. Altogether, these data support the notion that *B4GALNT2* is a key member of a gene signature associated with good prognosis. Amongst the genes differentially expressed in HBE and LBE, several are involved in glycosylation (Table 2). Differential expression of several glycosyltransferases predicts that cancer cells of HBE display higher levels of mucin-type *O*-glycosylation (*GALNT8*) with sugar chains terminating with sialyl-Tn, (*ST6GALNAC1*), sialyl-6-T structures (*ST6GALNAC2*), and Core 3 structures (*B3GNT6*); increased biosynthesis of type 1 chains (*B3GALT5*); and increased α2,3 sialylation of type 2 chains (*ST3GAL4*), forming acceptor substrates for B4GALNT2. Despite the higher expression of *FUT5*, more biosynthesis of sLe^x^ does not appear to be likely, considering the competition of B4GALNT2. Owing to the reduced expressions of both *ST6GAL1* and *ST6GAL2*, α2,6-sialylation could be reduced in HBE. Among non-glycosyltransferase molecules, it is worth mentioning the higher expression of galectin 4, which is associated with normal gut, and of the gel-forming mucins MUC2, MUC4, and MUC5B. These data support the existence of different glycophenotypes in the LBE and HBE cohorts.

Current data indicate that *B4GALNT2* control in colonic tissues consists of complex mechanisms. We showed that DNA-methylation plays an important, although unconventional, role (Figure 4). In fact, its general downregulation observed in the vast majority of cancer cases cannot be explained by differential methylation of CpG sites located on the island and shores. Rather, the open-sea site located at the intron between exons 6 and 7 presents generally reduced methylation in cancer samples. Interestingly, methylation of this site is associated with increased, rather than decreased, *B4GALNT2* expression. In some cases, the stimulatory effect of high methylation of specific sequences is due to the fact that it promotes interactions between distant enhancers and the regulatory regions of the gene [46]. However, regardless a permissive methylation status, in many samples, *B4GALNT2* was not expressed, indicating that other regulatory mechanisms are involved. This is in agreement with a previous study showing that differential *B4GALNT2* promoter methylation is only partially correlated with gene expression in gastric cancer cell lines [17]. miRNAs are potential candidates for *B4GALNT2* regulation. Among the miRNAs theoretically predicted to inhibit *B4GALNT2*, miR-204–5p appears to be the most plausible candidate (Figure 5). In fact, it is the only one downregulated in HBE and none of the samples expressing *B4GALNT2* above a background threshold expressed this miRNA. However, miR-204–5p downregulation does not ensure *B4GALNT2* expression, since many cases lacking miR-204–5p failed to express *B4GALNT2*. Together, these data indicate a multifactorial nature of *B4GALNT2* regulation, with DNA methylation and miRNA expression playing relevant but not exclusive roles. It appears that, even without methylation and miRNA “brakes”, the “engine” of *B4GALNT2* transcription does not run. The lack of appropriate transcription factors is a plausible reason for the lack of *B4GALNT2* expression in the majority of CRC samples. Our previous data indicate that forced *B4GALNT2* expression is responsible for attenuation of the neoplastic phenotype and of stemness in different CRC cell models [13,20]. On this basis, the stimulation of *B4GALNT2* expression in CRC cells can be proposed as a promising goal for therapy of this deadly disease.

## 4. Materials and Methods

Kaplan–Meier survival curves were obtained from the Oncolnc website (oncolnc.org), selecting the COAD cohort and the 15 higher and the 15 lower percentiles of gene expression. Gene expression data of 626 colorectal adenocarcinoma (COADREAD) samples were downloaded from the TCGA database using the Firebrowse website (http://firebrowse.org (accessed on 11 April 2021)). The gene methylation data of 288 tumor samples were downloaded from TCGA. A comparison with gene methylation between normal and cancer tissues was performed using the Smartapp tool (http://www.bioinfo-zs.com/smartapp/ (accessed on 11 April 2021)). A search for miRNAs targeting *B4GALNT2* was completed using the CSmiRTar website (http://cosbi4.ee.ncku.edu.tw/CSmiRTar/ (accessed on 11 April 2021)), selecting “cancer” as “class of disease”; “colorectal cancer” as “disease”, and “colorectum” as “tissue”. Only miRNA predicted by at least two databases were considered. The number of TCGA patients for which miRNA expression data were available varied for different miRNA from 221 and 298.

Patients were ordered according to increasing levels of *B4GALNT2* expression. Two cohorts (LBE and HBE) containing 94 patients falling in the 15% low percentile and 15% high percentile, respectively, were selected. Genes differentially modulated in the HBE and LBE cohorts were analyzed by the false discovery rate two-stage linear step-up procedure of Benjamini, Krieger, and Yekutieli. The role of the genes was deduced from the website “genecards.org”, while the role of the genes in cancer was deduced by an extensive PubMed search using the “gene name” and “cancer” as search terms.

## Figures and Tables

**Table 1:** Genes differentially modulated in HBE and LBE.

Gene	Ratio	Gene Role	Reference	
*CLCA1*	203	Involved in mucus secretion and as a tumor suppressor and suppresses CRC malignancy	28974231	
*ZG16*	151	Animal lectin; inhibits growth and increases immune surveillance of CRC	[26,27,28,29]	
*ITLN1*	62	Lectin-recognizing microbial carbohydrates; protective in CRC	[30,31,32]	
*CLCA4*	51	Involved in mediating chloride conductance; downregulated genes in CRC	32027181	
*SPINK4*	48	Serine peptidase inhibitor; its downregulation is associated with poor survival in CRC	31888570	
*CA1*	45	Carbonic anhydrase; predictive biomarker in CRC	32031891	
*MAGEA1*	37	Involved in transcriptional regulation; acts as an oncogene in some cancers	30509089	
*PYY*	33	Inhibitis intestinal mobility; decreased expression is associated with CRC	11825654	
*GUCA2B*	32	Regulator of intestinal fluid transport; tumor suppressor in CRC	29788743	
*CA4*	27	Stimulates the ion transporter activity of SLC4A4; Predictive biomarker in CRC	32031891	
*MS4A12*	25	Involved in signal transduction; promotes malignant progression in CRC	18451174	
*BEST2*	23	Anion channel; methylation marker for early detection and prognosis of CRC	[33]	
*HEPACAM2*	23	Required for centrosome maturation; associated with good prognosis	29659199	
*TMIGD1*	22	Controls cell–cell adhesion and proliferation; tumor suppressor in CRC	33129760	
*CLDN8*	16	Claudin 8; component of tight junctions; downregulated in CRC	21479352	
*B3GNT6*	14	Synthesizes core 3 O-linked chains; downregulation associated with malignancy in CRC	28745318	
*KIF19*	13	Microtubule-dependent motor protein; higher expression associated with longer survival	28901309	
*CSAG2*	13	Chondrosarcoma-asociated gene 2/3 protein; necessary for tumorigenesis	32761762	
*FCGBP*	12	Maintains the mucosal structure; high expression is associated with better prognosis	31268166	
*CDKN2BAS*	12	CDKN2B antisense RNA 1; promotes progression of ovarian cancer	32572907	
*REG1B*	11	Regenerating islet-derived protein 1-β; its silencing inhibits CRC growth	25768000	
*IGJ*	11	Joining chain of multimeric IgA and IgM; downregulated in CRC	31749922	
*LEFTY2*	10	Member of the TGF-β superfamily; negative regulator of endometrial cell proliferation	27497669	
*FUT5*	10	Fucosyltransferase 5; promotes the development of CRC	28771224	
*MUC2*	10	Secreted mucus-forming mucin; suppresses CRC migration and metastasis	28725043	
*PLIN1*	−4	Modulator of adipocyte lipid metabolism; inhibits breast cancer cell proliferation	27359054	
*PCP4*	−4	Functions as a modulator of calcium-binding by calmodulin; antiapoptotic peptide	25153723	
*IGF2*	−4	Possess growth-promoting activity; overexpression is associated with poor prognosis	24080445	
*SLC14A1*	−4	Urea channel; cancer stem cell marker	29329541	
*FREM1*	−5	Extracellular matrix protein; associated with better prognosis in bladder cancer	33058542	
*CASQ2*	−5	Calsequestrin; high expression associated with poor survival in bladder cancer	31991631	
*CPLX2*	−6	Involved in exocytosis; associated with poor prognosis in lung tumors	3912489	
*ADIPOQ*	−6	Adiponectin; anti-inflammatory adipokine; lower expression in CRC	27061803	
*WIF1*	−7	Inhibits WNT actvities; hypermethylation is associated with a favorable clinical outcome	31830937	
*CHRNB2*	−9	Cholinergic receptor nicotinic beta 2 subunit; downregulated in gastric cancer	30175534	
*AP3B2*	−10	Involved in protein sorting; low expression is associated with long-term survival in rectal cancer	29050227	
*TSIX*	−11	XIST antisense RNA; dysregulates cancer pathways in multiple tumor contexts	[34]	

Genes differentially modulated in HBE and LBE cohorts were analyzed by the false discovery rate two-stage linear step-up procedure of Benjamini, Krieger, and Yekutieli. Only genes showing upregulation ≥ 10 or downregulation ≤ −4 and with a recognized role in cancer, as deduced by the literature, are reported. Mean level of expression ± SD and corrected *p* values are reported in Table S1. “Ratio” refers to the HBE/LBE ratio. When the expression was higher in LBE, the HBE/LBE ratio was expressed preceded by a “minus” sign. The role of the gene was deduced from the website “genecards.org”, whereas the role of the gene in cancer was deduced by an extensive literature search. The “Reference” column reports either the number in the Reference list or the PubMed accession number. The red or green labels indicate putative tumor-promoting or tumor-restraining changes, respectively.

**Table 2:** Expression level of glycogenes in HBE and LBE.

Functional Class	Gene	Ratio	Role
First steps of *O*-linked biosynthesis	*GALNT8*	9.5	Addition of the first GalNAc residue of the *O*-linked chains
	*B3GNT6*	14.4	Synthesis of Core 3 *O*-glycans by attaching GlcNAc to GalNAc
	*ST6GALNAC1*	5.4	Synthesis of sialyl Tn by attaching Sia to GalNAc
	*ST6GALNAC2*	2.1	Synthesis of sialyl T by attaching Sia to GalNAc of T antigen
Ganglioside biosynthesis	*ST6GALNAC6*	2.5	Synthesis of higher gangliosides
Proteoglycan biosynthesis	*B3GNT7*	6.3	Keratan sulfate biosynthesis
Biosynthesis of sialyl Lewis antigens	*B3GALT5*	4.0	Synthesis of type 1 chains
	*ST3GAL4*	4.0	Sialylation of type 2 chains
	*FUT5*	10.8	Fucosylation of type 2 chains
Biosynthesis of Sia6 LacNAc structures	*ST6GAL1*	−2.0	α2,6 sialylation of glycoproteins
	*ST6GAL2*	−2.7	α2,6 sialylation of soluble substrates
Galactose recognition	*LGALS4*	2.0	Galectin 4, expressed in the gut, underexpressed in CRC
	*LGALS9B*	2.2	Highly similar to Galectin 9
*O*-glycoproteins	*MUC1*	2.0	Membrane bound mucin with multiple functions
	*MUC2*	10.4	Secreted mucus forming mucin
	*MUC4*	5.2	Membrane and secreted mucin
	*MUC5B*	2.6	Gel-forming mucin

The role of glycosyltransferases is indicated in Supplementary Figure S2. Mean level of expression ± SD and corrected *p*-values are reported in Table S1. “Ratio” indicates the ratio between gene expression in HBE/LBE. When the expression was higher in LBE, the HBE/LBE ratio was expressed with a “minus” sign.

## Data Availability

Not applicable.

## Supplementary Materials

The following are available online at https://www.mdpi.com/article/10.3390/ijms22094331/s1.

## Abbreviations

COADREAD	colon adenocarcinoma rectal adenocarcinoma
CRC	colorectal cancer
HBE	higher B4GALNT2 expressers
LBE	lower B4GALNT2 expressers
lncRNA	long noncoding RNA
NMR	normalized miRNA score
PD-L1	programmed death ligand-1
sLe^x^	Sialyl Lewis^X^
TCGA	The Cancer Genome Atlas

## References

[1] Varki A. (2017). Biological roles of glycans. Glycobiology.

[2] Dall’Olio F., Malagolini N., Trinchera M., Chiricolo M. (2012). Mechanisms of cancer-associated glycosylation changes. Front. Biosci..

[3] Dall’Olio F., Malagolini N., Trinchera M., Chiricolo M. (2014). Sialosignaling: Sialyltransferases as engines of self-fueling loops in cancer progression. Biochim. Biophys. Acta.

[4] Pinho S.S., Reis C.A. (2015). Glycosylation in cancer: Mechanisms and clinical implications. Nat. Rev. Cancer.

[5] Dall’Olio F., Malagolini N., Chiricolo M., Trinchera M., Harduin-Lepers A. (2014). The expanding roles of the Sd^a^/Cad carbohydrate antigen and its cognate glycosyltransferase B4GALNT. Biochim. Biophys. Acta.

[6] Serafini-Cessi F., Dall’Olio F. (1983). Guinea-pig kidney β-N-acetylgalactosaminyltransferase towards Tamm-Horsfall glycoprotein. Requirement of sialic acid in the acceptor for transferase activity. Biochem. J..

[7] Lo Presti L., Cabuy E., Chiricolo M., Dall’Olio F. (2003). Molecular Cloning of the Human β1,4 N-Acetylgalactosaminyltransferase Responsible for the Biosynthesis of the Sd^a^ Histo-Blood Group Antigen: The Sequence Predicts a Very Long Cytoplasmic Domain. J. Biochem..

[8] Montiel M.D., Krzewinski-Recchi M.A., Delannoy P., Harduin-Lepers A. (2003). Molecular cloning, gene organization and expression of the human UDP-GalNAc:Neu5Acα2-3Galβ-R β1,4-N-acetylgalactosaminyltransferase responsible for the biosynthesis of the blood group Sda/Cad antigen: Evidence for an unusual extended cytoplasmic domain. Biochem. J..

[9] Smith P.L., Lowe J.B. (1994). Molecular cloning of a murine N-acetylgalactosamine transferase cDNA that determines expression of the T lymphocyte-specific CT oligosaccharide differentiation antigen. J. Biol. Chem..

[10] Stenfelt L., Hellberg A., Moller M., Thornton N., Larson G., Olsson M.L. (2019). Missense mutations in the C-terminal portion of the *B4GALNT2*-encoded glycosyltransferase underlying the Sd(a-) phenotype. Biochem. Biophys. Rep..

[11] Malagolini N., Dall’Olio F., Di Stefano G., Minni F., Marrano D., Serafini-Cessi F. (1989). Expression of UDP-GalNAc:NeuAc α2,3Gal β-R beta 1,4(GalNAc to Gal) N-acetylgalactosaminyltransferase involved in the synthesis of Sd^a^ antigen in human large intestine and colorectal carcinomas. Cancer Res..

[12] Malagolini N., Santini D., Chiricolo M., Dall’Olio F. (2007). Biosynthesis and expression of the Sd^a^ and sialyl Lewis x antigens in normal and cancer colon. Glycobiology.

[13] Pucci M., Gomes Ferreira I., Orlandani M., Malagolini N., Ferracin M., Dall’Olio F. (2020). High Expression of the Sd^a^ Synthase B4GALNT2 Associates with Good Prognosis and Attenuates Stemness in Colon Cancer. Cells.

[14] Staubach F., Kunzel S., Baines A.C., Yee A., McGee B.M., Backhed F., Baines J.F., Johnsen J.M. (2012). Expression of the blood-group-related glycosyltransferase B4galnt2 influences the intestinal microbiota in mice. ISME J..

[15] Groux-Degroote S., Schulz C., Cogez V., Noel M., Portier L., Vicogne D., Solorzano C., Dall’Olio F., Steenackers A., Mortuaire M. (2018). The extended cytoplasmic tail of the human B4GALNT2 is critical for its Golgi targeting and post-Golgi sorting. FEBS J..

[16] Wang H.R., Hsieh C.Y., Twu Y.C., Yu L.C. (2008). Expression of the human Sd^a^ β-1,4-N-acetylgalactosaminyltransferase II gene is dependent on the promoter methylation status. Glycobiology.

[17] Kawamura Y.I., Toyota M., Kawashima R., Hagiwara T., Suzuki H., Imai K., Shinomura Y., Tokino T., Kannagi R., Dohi T. (2008). DNA hypermethylation contributes to incomplete synthesis of carbohydrate determinants in gastrointestinal cancer. Gastroenterology.

[18] Kawamura Y.I., Kawashima R., Fukunaga R., Hirai K., Toyama-Sorimachi N., Tokuhara M., Shimizu T., Dohi T. (2005). Introduction of Sd^a^ carbohydrate antigen in gastrointestinal cancer cells eliminates selectin ligands and inhibits metastasis. Cancer Res..

[19] Kawamura Y.I., Adachi Y., Curiel D.T., Kawashima R., Kannagi R., Nishimoto N., Dohi T. (2014). Therapeutic adenoviral gene transfer of a glycosyltransferase for prevention of peritoneal dissemination and metastasis of gastric cancer. Cancer Gene Ther..

[20] Pucci M., Gomes F.I., Malagolini N., Ferracin M., Dall’Olio F. (2020). The Sd^a^ Synthase B4GALNT2 Reduces Malignancy and Stemness in Colon Cancer Cell Lines Independently of Sialyl Lewis X Inhibition. Int. J. Mol. Sci..

[21] Jung B., Staudacher J.J., Beauchamp D. (2017). Transforming Growth Factor beta Superfamily Signaling in Development of Colorectal Cancer. Gastroenterology.

[22] Cheasley D., Pereira L., Sampurno S., Sieber O., Jorissen R., Xu H., Germann M., Yuqian Y., Ramsay R.G., Malaterre J. (2015). Defective Myb Function Ablates Cyclin E1 Expression and Perturbs Intestinal Carcinogenesis. Mol. Cancer Res..

[23] Noda M., Okayama H., Tachibana K., Sakamoto W., Saito K., Thar Min A.K., Ashizawa M., Nakajima T., Aoto K., Momma T. (2018). Glycosyltransferase gene expression identifies a poor prognostic colorectal cancer subtype associated with mismatch repair deficiency and incomplete glycan synthesis. Clin. Cancer Res..

[24] Shimodaira K., Nakayama J., Nakamura N., Hasebe O., Katsuyama T., Fukuda M. (1997). Carcinoma-associated expression of core 2 β1,6-N-acetylglucosaminyltransferase gene in human colorectal cancer: Role of O-glycans in tumor progression. Cancer Res..

[25] An G., Wei B., Xia B., McDaniel J.M., Ju T., Cummings R.D., Braun J., Xia L. (2007). Increased susceptibility to colitis and colorectal tumors in mice lacking core 3-derived O-glycans. J. Exp. Med..

[26] Meng H., Li W., Boardman L.A., Wang L. (2018). Loss of ZG16 is associated with molecular and clinicopathological phenotypes of colorectal cancer. BMC. Cancer.

[27] Meng H., Ding Y., Liu E., Li W., Wang L. (2021). ZG16 regulates PD-L1 expression and promotes local immunity in colon cancer. Transl. Oncol..

[28] Meng Q., Ren C., Wang L., Zhao Y., Wang S. (2015). Knockdown of ST6Gal-I inhibits the growth and invasion of osteosarcoma MG-63 cells. Biomed. Pharmacother..

[29] Mito A., Nakano Y., Saitoh T., Gouraud S.S.S., Yamaguchi Y., Sato T., Sasaki N., Kojima-Aikawa K. (2018). Lectin ZG16p inhibits proliferation of human colorectal cancer cells via its carbohydrate-binding sites. Glycobiology.

[30] Liu J., Jiang C., Xu C., Wang D., Shen Y., Liu Y., Gu L. (2021). Identification and development of a novel invasion-related gene signature for prognosis prediction in colon adenocarcinoma. Cancer Cell Int..

[31] Kawashima K., Maeda K., Saigo C., Kito Y., Yoshida K., Takeuchi T. (2017). Adiponectin and Intelectin-1: Important Adipokine Players in Obesity-Related Colorectal Carcinogenesis. Int. J. Mol. Sci..

[32] Liu X., Bing Z., Wu J., Zhang J., Zhou W., Ni M., Meng Z., Liu S., Tian J., Zhang X. (2020). Integrative Gene Expression Profiling Analysis to Investigate Potential Prognostic Biomarkers for Colorectal Cancer. Med. Sci. Monit..

[33] Qin L., Zeng J., Shi N., Chen L., Wang L. (2020). Application of weighted gene coexpression network analysis to explore the potential diagnostic biomarkers for colorectal cancer. Mol. Med. Rep..

[34] Chiu H.S., Somvanshi S., Patel E., Chen T.W., Singh V.P., Zorman B., Patil S.L., Pan Y., Chatterjee S.S., Sood A.K. (2018). Pan-Cancer Analysis of lncRNA Regulation Supports Their Targeting of Cancer Genes in Each Tumor Context. Cell Rep..

[35] Holst S., Wuhrer M., Rombouts Y. (2015). Glycosylation characteristics of colorectal cancer. Adv. Cancer Res..

[36] Capon C., Maes E., Michalski J.C., Leffler H., Kim Y.S. (2001). Sd^a^-antigen-like structures carried on core 3 are prominent features of glycans from the mucin of normal human descending colon. Biochem. J.

[37] Salvini R., Bardoni A., Valli M., Trinchera M. (2001). β1,3-Galactosyltransferase β3Gal-T5 acts on the GlcNAcβ1-->3Galβ1-->4GlcNAcβ1-->R sugar chains of carcinoembryonic antigen and other N-linked glycoproteins and is down-regulated in colon adenocarcinomas. J Biol. Chem..

[38] Weinstein J., Lee E.U., McEntee K., Lai P.H., Paulson J.C. (1987). Primary structure of β-galactoside α 2,6-sialyltransferase. Conversion of membrane-bound enzyme to soluble forms by cleavage of the NH_2_-terminal signal anchor. J. Biol. Chem..

[39] Takashima S., Tsuji S., Tsujimoto M. (2002). Characterization of the Second Type of Human β-Galactoside α2,6-Sialyltransferase (ST6Gal II), Which Sialylates Galβ1,4GlcNAc Structures on Oligosaccharides Preferentially. Genomic Analysis of Human Sialyltransferase Genes. J Biol. Chem..

[40] Dall’Olio F., Malagolini N., Di Stefano G., Minni F., Marrano D., Serafini-Cessi F. (1989). Increased CMP-NeuAc:Galβ1,4GlcNAc-R α 2,6 sialyltransferase activity in human colorectal cancer tissues. Int. J. Cancer.

[41] Lise M., Belluco C., Perera S.P., Patel R., Thomas P., Ganguly A. (2000). Clinical correlations of α2,6-sialyltransferase expression in colorectal cancer patients. Hybridoma.

[42] Venturi G., Gomes F.I., Pucci M., Ferracin M., Malagolini N., Chiricolo M., Dall’Olio F. (2019). Impact of sialyltransferase ST6GAL1 overexpression on different colon cancer cell types. Glycobiology.

[43] Mito A., Kumazawa-Inoue K., Kojima-Aikawa K. (2020). ZG16p, an Animal Homologue of Plant beta-Prism Fold Lectins: Purification Methods of Natural and Recombinant ZG16p and Inhibition Assay of Cancer Cell Growth Using ZG16p. Methods Mol. Biol..

[44] Pattison A.M., Merlino D.J., Blomain E.S., Waldman S.A. (2016). Guanylyl cyclase C signaling axis and colon cancer prevention. World J. Gastroenterol..

[45] Low E.N.D., Mokhtar N.M., Wong Z., Raja Ali R.A. (2019). Colonic Mucosal Transcriptomic Changes in Patients with Long-Duration Ulcerative Colitis Revealed Colitis-Associated Cancer Pathways. J. Crohns. Colitis..

[46] Grimmer M.R., Costello J.F. (2016). Cancer: Oncogene brought into the loop. Nature.

