# Supplementary material for: Glycosyltransferase B4GALNT2 as a Predictor of Good Prognosis in Colon Cancer: Lessons from Databases
Source: Int J Mol Sci. 2021 Apr 21;22(9):4331. doi: 10.3390/ijms22094331 (PMC8122605; doi:10.3390/ijms22094331)
Supplement: Supplementary file 1 [file ijms-22-04331-s001.zip › ijms-1184321-supplementary/ijms-1184321-supplementary-proof done/Supplementary Figures.docx]

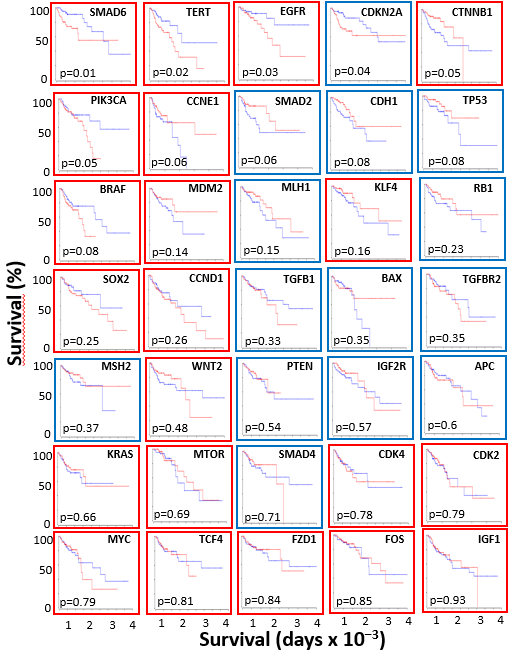


Figure S1. Kaplan Meier survival curves of patients expressing different levels of oncogenes and tumor-suppressor genes. Curves were generated by the OncoLnc.org site using the 15% higher (red lines) and 15% lower expressers (blu lines) of the indicated genes. Graphs have been ordered according to the increasing p value and boxed in red or blue according to the recognized role as tumor promoting or tumor suppressing activity of the gene.


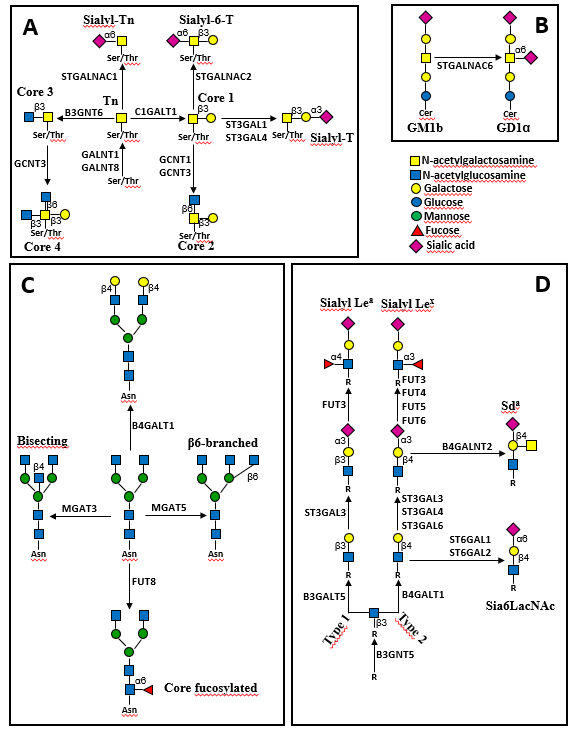


Figure S2. Carbohydrate structures and glycosyltransferases involved in their biosynthesis.


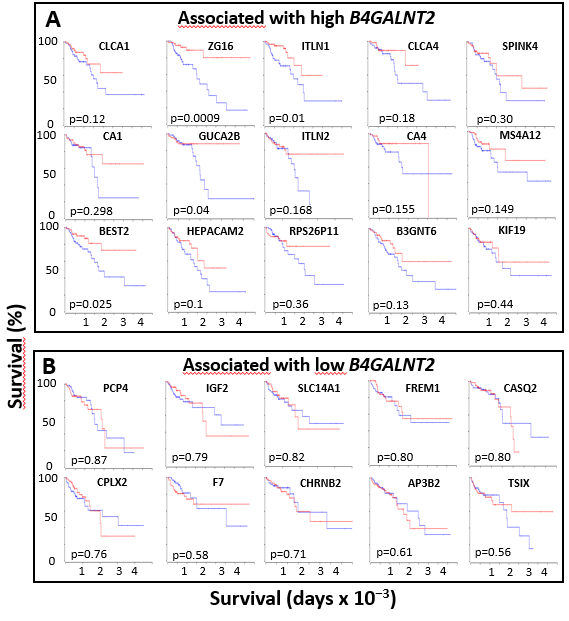


Figure S3. Kaplan Meier survival curves of patients expressing different levels of genes in the LBE and HBE cohorts. Curves were generated using the 15% higher (red lines) and 15% lower expressers (blu lines) of the indicated genes. A: 15 top highly up-regulated genes in HBE (from Table S1). The following genes are not reported in the COAD cohort: *MAGEA1, PYY, TMIGD1, HSD3B2; FAM5C (BRINP3), CLDN8*. B: 10 top down-regulated genes in HBE (Table S1) . The following genes are not reported in the COAD cohort: *WIF1, ADIPOQ*.
